# Sensor Reliability Evaluation Scheme for Target Classification Using Belief Function Theory

**DOI:** 10.3390/s131217193

**Published:** 2013-12-13

**Authors:** Jing Zhu, Yupin Luo, Jianjun Zhou

**Affiliations:** 1 Tsinghua National Laboratory for information Science and Technology (TNList), Department of Automation, Tsinghua University, Beijing 100084, China; E-Mail: luo@tsinghua.edu.cn; 2 Navy Academy of Armament, Beijing 102249, China; E-Mail: yuqiaozhou@126.com; 3 Bejing University of Aeronautics and Astronautics, Beijing 100191, China

**Keywords:** information fusion, sensor reliability, belief function theory, discounting factor, target classification

## Abstract

In the target classification based on belief function theory, sensor reliability evaluation has two basic issues: reasonable dissimilarity measure among evidences, and adaptive combination of static and dynamic discounting. One solution to the two issues has been proposed here. Firstly, an improved dissimilarity measure based on dualistic exponential function has been designed. We assess the static reliability from a training set by the local decision of each sensor and the dissimilarity measure among evidences. The dynamic reliability factors are obtained from each test target using the dissimilarity measure between the output information of each sensor and the consensus. Secondly, an adaptive combination method of static and dynamic discounting has been introduced. We adopt Parzen-window to estimate the matching degree of current performance and static performance for the sensor. Through fuzzy theory, the fusion system can realize self-learning and self-adapting with the sensor performance changing. Experiments conducted on real databases demonstrate that our proposed scheme performs better in target classification under different target conditions compared with other methods.

## 1. Introduction

Belief function theory has been widely applied in intelligent decision systems [1], which is obviously influential in the representation, measure and combination of uncertainty. In the multisensor information fusion process, the output of each sensor is assigned the same reliability in the Dempster rule of combination [1]. In fact, each sensor has different capacity, so it is not reasonable to keep reliability constant for each sensor, especially for heterogeneous sensors (such as optical sensors, RADAR and infrared sensors). Firstly, evaluating the reliability of sensors accurately and amending output evidence are necessary to improve the robustness of fusion systems and decrease the side effects of sensor output with evidence of low reliability. Secondly, the distinction of the sensors' reliability is an important factor causing conflicts among evidences. By computing the reliability of each sensor, modifying the corresponding evidence is another important way of dealing with high conflicting evidences' combination. Thirdly, the reliability of sensors is closely related to the environment, which may change at any time. For example, some contextual factors often affect the reliability of sensors, such as target and background properties (environmental noise, and deceptive behaviors of observed targets) [2]. If the evaluation method for sensors' reliability is not adaptive to the environment and lacks self-learning ability, large deviations will occur in the fusion results. Therefore, correcting disadvantages in evidence modeling, adapting to different environments, and adding the reliability evaluation of sensors in the fusion process can solve problems of combining conflicting evidences [3–5].

The main purpose for the sensors' reliability evaluation is to determine an appropriate discounting factor for the sensor. We adopt the sensor discounting factor to denote its reliability according to the relation that the reliability is equal to 1 minus the discount factor [6], which includes static and dynamic reliability evaluation. The static reliability evaluation is based on the training samples and obtained the prior knowledge actually. The dynamic reliability is calculated in the test process without using the training sets and reflects performance changes of sensor. That is to say, the static reliability is prior information while the dynamic reliability is real-time information. These two kinds of reliability factors can be combined together. The overall framework, which relates to the problem of general fusion of uncertainty information, was originally put forward by Rogova [7]. In [6], Elouedi evaluated the reliability of sensors with the transferable belief model. Then, Guo [2] improved the acquisition scheme of the reliability factor and presents the application strategy under the belief function theory framework. To acquire the sensors' reliability factors, Yang [8] combined the sensor confusion matrix of *a priori* static information and dynamic information of current output decisions. Elouedi [9] determined the static reliability factor of sensors by correcting the recognition rate on all training samples. Delmotte and Gacquer [10] proposed one mechanism of handling conflicts to detect defective sources, and designed the time-varying combination of dynamic reliability and static reliability. Other static or dynamic reliability evaluation methods are presented in [11–13].

After obtaining the reliability factor, basic belief assignment (BBA) from a multisensor can be corrected by the corresponding factors. The most classic method is Shafer's discounting rule [1]. Mercier [14] has proposed contextual discounting based on classic discounting, and gave the mathematical derivation process. Denoeux and Smets analyzed the inverse operation of discounting, that is, de-discounting in the classification issue [15]. Other examples of discounting work on multisensors can be found in [16,17]. Due to the high computational complexity of the contextual discounting method, this paper applies Shafer's discounting rule.

However, the existing methods of sensor reliability evaluation and evidence discounting have several problems. The dissimilarity measure of reasonable evidences is the basic issue of both static and dynamic reliability assessment. For example, dissimilarity measures among evidences are unreasonable in Guo's [2] and Elouedi's method [6]. Moreover, some methods use information inadequately, such as Elouedi's *T_f_* [9] and Yang's method [8]. In addition, the research on methods of combining static and dynamic discounting factors is not deep enough, which is just mentioned in [2]. This combination method has no ability to adapt to the performance changes of sensors.

In order to resolve the above problems, this paper puts forward a scheme of sensor reliability evaluation and evidence discounting, which mainly includes two parts. First, we have designed an improved dissimilarity measure based on a dualistic exponential function so as to assess the static reliability from a training set by local decision of each sensor and distance measure between evidences. The dynamic reliability factors are gained from every test target by dissimilarity measures between the output information of each sensor and the consensus of total evidences simultaneously. Second, we have introduced an adaptive combination method of static and dynamic discounting based on fuzzy theory and Parzen-window density estimation, which can be suitable for different kinds of uncertain target environments.

The rest of the paper is divided into six parts. Section 2 reviews the belief function theory. An improved dissimilarity measure based on a dualistic exponential function is presented in Section 3. Evaluation methods of static and dynamic discounting factor are respectively introduced in Section 4. In Section 5, we propose an adaptive combination mechanism of static and dynamic reliability discounting. The experiments and analysis are arranged in Section 6, where we compare the proposed method with other methods on real datasets. Then, a conclusion is presented in Section 7.

## 2. Basic Concepts of the Belief Function Theory

Belief function theory is regarded as a useful tool of representing and processing uncertain knowledge. In this section, a brief review of the belief function theory is introduced.

### 2.1. Main Function

Let Ω = {*ω_1_*, *ω_2_*, …, *ω_p_*} be a finite set of all possible results to a given problem, which is named as the frame of discernment. All the elements of Ω are exclusive and exhaustive, and belong to the power set of Ω, denoted as 2^Ω^. The subsets of Ω containing only one element are called singletons.

*Definition 1:* Given a set of evidence provided by the sensor, intelligent agent defines the corresponding basic belief assignment on Ω as a function *m*^Ω^:2^Ω^ → [0,1], which satisfies:

(1) $$\sum_{A\subseteq \Omega }m^{\Omega }\left(A\right)=1$$

If there is no ambiguity, *m*^Ω^ may be abbreviated to *m*. ∀*x* ⊆ *Ω*, the value of *m*(*x*) is called the basic belief mass (BBM), representing the belief portion of an agent *Ag* committed exactly to the proposition A, and nothing is more specific. The subsets *x* of Ω with a property *m*(*x*) > 0 are called focal elements of BBA *m*.

The mass *m*(Ω) represents the degree of ignorance of agent *Ag*. When *m*(Ω) = 1, *m* is called a vacuous BBA, which corresponds to complete ignorance of *x*'s value. The value *m*(*ϕ*) is the conflictive degree, and *m*(*ϕ*) = 0 is not necessarily required; and if it is not, this corresponds to the open-world assumption. A certain BBA expresses the total certainty.

*Definition 2:* Let BBA *m* be defined on a frame of discernment Ω; the belief function and the plausibility function are defined respectively as follows:

(2) $$\text{Bel}\;\left(A\right)=\sum_{B\subseteq A}m\left(B\right)\;\text{for}\;A\subseteq \Omega$$

(3) $$Pl\;\left(A\right)=\sum_{B\subseteq A\neq \phi }m\left(B\right)=1-\text{Bel}\left(A^{c}\right)$$

The belief function *Bel* is a measure of the total belief committed to *A* without being committed to *_Ā_*. The plausibility function *Pl* quantifies the maximum amount of belief that could be given to a subset *A* of Ω. Three functions above are in one-to-one correspondence.

### 2.2. Combination

The combination of multiple BBAs can be realized through the conjunction rule. Let *m*_1_ and *m*_2_ be two BBAs which are induced from two distinct pieces of evidence. Based on the closed-world assumption, two BBAs' conjunctive combination, denoted *m*_1⊕2_, for all the *A* ⊆ *Ω* are defined as follows:

(4) $$m_{1⊕2}\;\left(A\right)=C_{12}\sum_{\begin{array}{c} B\cap C=A\\ B,C\subseteq \Omega \end{array}}m_{1}\left(B\right)m_{2}\left(C\right)$$

The normalized factor is:

(5) $$C_{12}=1/\left[1-\sum_{\begin{array}{c} B\cap C=\phi \\ B,C\subseteq \Omega \end{array}}m_{1}\left(B\right)m_{2}\left(C\right)\right]$$

where the term *C*_12_ is called the conflict between *m*_1_ and *m*_2_, and it may be regarded as a simple measure of dissimilarity between BBAs. The conjunctive rules are both commutative and associative.

### 2.3. Classical Discounting

Because of the various conditions' influence, doubts about the reliability of an information source *m* are sometimes possible. Assuming that a source has probability (1 − *α*) of reliability (0 ≤ *α* ≤ 1), the discounting operation [1] of *m* has been named discounting rate. This correction operation of *m* is define as:

(6) $$\left\{ \begin{array}{ll} m^{\alpha }\;(A)=(1-\alpha )m(A) & \forall A\subset \Omega \\ m^{\alpha }\;(A)=(1-\alpha )m(A)+\alpha & if\;A=\Omega \end{array}\right.$$

The probability of reliability (1 − *α*) represents the source's reliability degree. If the source is completely unreliable, this degree equals to 0, then *α* is equal to 1. On the contrary, if the source is absolutely reliable, then *α* equals to 0, and *m* will not be discounted. Other mechanisms of discounting are presented in [14–16,18].

### 2.4. Pignistic Transformation

In the transferable belief model (TBM) [19], pignistic probabilities are used for making decisions. The transferable belief model is based on two levels:

- The credal level and its beliefs are expressed by belief functions.
- In the pignistic level where for the purpose of making decisions, belief functions are converted into the pignistic probabilities denoted *BetP*.

The relation can be constructed between the two functions by the pignistic transformation on Ω:

(7) $$\text{BetP}\left(A\right)=\sum_{B\subseteq \Omega }\frac{\left|A\cap B\right|}{\left|B\right|}\frac{m\left(B\right)}{1-m\left(\phi \right)},\forall A\subseteq \Omega .$$

## 3. An Improved Dissimilarity Measure about BBAs

### 3.1. Problem Description

Fundamentally speaking, an accurate dissimilarity measure between BBAs is the basis of the sensor reliability evaluation, either static or dynamic. For instance, the basic idea of the dynamic discounting method is that, if one source of evidence is different from other sources, it has a low reliability.

In this section, we first review the existing dissimilarity measure methods used in sensor reliability evaluation. Then, an improved dissimilarity measure method based on dualistic exponential function has been designed. Finally, several dissimilarity measure methods are compared and discussed.

### 3.2. The Existing Dissimilarity Measure Methods

In belief function theory, the dissimilarity between evidences reflects the inconsistency of sensors. In order to describe the inconsistency in a quantity, it is necessary to define quantitatively the dissimilarity measure, then, the target-oriented corresponding strategy emerges in such circumstances. There are three dissimilarity measure methods, namely BBM type, distance type and complex type dissimilarity measures.

#### 3.2.1. BBM Type of Dissimilarity Measure

This was been proposed by Shafer [1]. BBM is given to the empty set in the process of conjunction combination rule. However, the BBM cannot be committed frequently owing to the counterintuitive problem. Based on Shafer's work, Jia [13] has proposed a generalized dissimilarity measure, and considered the common effects of direct conflict and potential conflict.

#### 3.2.2. Distance Type of Dissimilarity Measure

The evidences are regarded as linear space vectors in this dissimilarity measure, which reflects the geometric meaning of the inconsistency between evidences. On the basis of distance metric definition [20], this dissimilarity measure contains the specific mathematical format. Such methods include Wang's distance measure [21], Jousselme's distance measure [22], and He's distance measure [23], *etc*.

#### 3.2.3. Complex Type of Dissimilarity Measure

This dissimilarity measure combines both the BBM type and distance type measures, and has the form of dualistic variables corresponding to two kinds of measures [20,24]. The expressions, advantages, and disadvantages for the above three kinds of dissimilarity measure methods are shown in Table 1 (formulas and symbols follow the definitions of Section 2, and *K* is the number of evidences).

### 3.3. Our Dissimilarity Measure Method

Before designing the measurement method, the dissimilarity measurement should be firstly fit for the intuitive logic of persons. Secondly, it can measure the dissimilarity among more than two pieces of evidence simultaneously. In addition, the dissimilarity measurement should have good operational capability. Considering the comprehensiveness of dissimilarity measurement, this paper integrates both the BBM type and distance type dissimilarity measurements. Firstly, the new function is proposed to replace *C*_1…_*_K_*, and then a new measurement form is designed for more than three pieces of evidence, which overcomes the *difBetP* problem. With the certain one-sidedness of Guo's method and poor maneuverability of Liu's method, this paper uses the binary function form. In the compromise process, our paper represents evidence measurement by using an improved binary function.

The specific strategy includes three steps:

#### 3.3.1. Improvement of *C*_1…_*_K_*

The problem of *C*_1…_*_K_* is that the conflict measure results are often counterintuitive. Based on different consistency measure functions [25,26] in Table 2, this paper adopts the form of the Dice measure function.

This paper expounds that the local dissimilarity consists of local potential dissimilarity and local direct dissimilarity. We construct the local potential dissimilarity among *K* pieces of evidences.

For *K* pieces of evidences corresponding BBA *m_k_*(*k* ∈ {1,2,…, *K*}) on the same discernment frame Ω, BBAs is given the BBM *m_k_*(*A_k_*) > 0 (*k* =1,2,…, *K* ) respectively on sets *A_1_*, *A_2_*,…*A_k_*. ‖*_k_*_=1_*A* ≠ *ϕ* and max {|*A_1_*|, |*A_2_*|,…|*A_k_*|} ≥ 2,the local potential dissimilarity among *K* pieces of evidence is:

(8) $$\xi_{A_{1},A_{2},\cdots ,A_{k}}=\left(1-\frac{K\times \left|\cap_{k=1}^{K}A_{k}\right|}{\sum_{k=1}^{K}\left|A_{k}\right|}\right)\prod_{k=1}^{K}{\text{m}}_{k}\left(A_{k}\right)$$

the local direct dissimilarity:

(9) $$\prod_{\begin{array}{c} k=1\\ \cap_{k=1}^{K}X_{k}=\phi \end{array}}^{K}m_{k}\left(X_{k}\right)$$

*Definition 3*: For *K* pieces of evidences corresponding to the BBA *m_k_*(*k* ∈ {1,2,…, *K*}) on the same discernment frame Ω, the total dissimilarity is defined as the sum of the local potential dissimilarity and the local direct dissimilarity between K pieces of evidences, namely:

(10) $$D_{1\cdots K}=\sum_{\cap_{k=1}^{K}A_{k}\neq \phi }\xi_{A_{1},A_{2},\cdots ,A_{k}}+\sum_{\cap_{k=1}^{K}A_{k}=\phi }\prod_{k=1}^{K}m_{k}\left(X_{k}\right)$$

Theorem 1: For *K* pieces of evidences corresponding to BBA *m_k_*(*k* ∈ {1,2,…, *K*}) on the same discernment frame Ω, the total dissimilarity is:

(11) $$D_{1\cdots K}=\sum_{A_{k}\subseteq \Omega }\left\{\left(1-\frac{K\times \left|\cap_{k=1}^{K}A_{k}\right|}{\sum_{k=1}^{K}\left|A_{k}\right|}\right)\prod_{k=1}^{K}m_{k}\left(A_{k}\right)\right\}$$

Proof: in Equation (8), we can see that:

If $\cap_{k=1}^{K}A_{k}=\phi$, then $\xi_{A_{1},A_{2},\cdots A_{k}}=\prod_{k=1}^{K}m_{k}(A_{k})$, local potential dissimilarity becomes local direct dissimilarity;

If $\cap_{k=1}^{K}A_{k}\neq \phi$, and |*A_1_*| = |*A_2_*| = |*A_k_*| = 1, then *ξ_A1_*,*_A2_*_,…_*_Ak_* = 0, and there is no dissimilarity.

Hence, whether local direct dissimilarity or local potential dissimilarity can be expressed by Equation (8). The total dissimilarity is the sum of local potential dissimilarity and local direct dissimilarity between evidences, then we can get:

$$D_{1\cdots K}=\sum_{A_{k}\subseteq \Omega }\xi_{A_{1},A_{2},\cdots A_{K}}=\sum_{A_{k}\subseteq \Omega }\left\{\left(1-\frac{K\times \left|\cap_{k=1}^{K}A_{k}\right|}{\sum_{k=1}^{K}\left|A_{k}\right|}\right)\prod_{k=1}^{K}m_{k}\left(A_{k}\right)\right\}$$

Theorem 2: For *D*_1…_*_k_* of Equation (11), there is always 0 ≤ *D*_1…_*_k_* ≤ 1.

Proof: on one hand, in Equation (11), we suppose $\left|\cap_{k=1}^{K}A_{k}\right|=x$, then there will be |*A_k_*| ≥ *x*, so we get $\sum_{k=1}^{K}\left|A_{k}\right|\geq Kx$, thus:

$$1-\frac{K\times |\cap_{k=1}^{K}A_{k}|}{\sum_{k=1}^{K}\left|A_{k}\right|}\geq 1-\frac{K\times x}{K\times x}=0$$

With *m_k_* (*A_k_*) ≥ 0, we get *D*_1…_*_k_* ≥ 0. On the other hand, we can get:

$$\begin{array}{ll} D_{1\cdots K} & =\underset{A_{k}\subseteq \Omega ,\cap A_{k}=\phi }{\text{∑}}\prod_{k=1}^{K}m_{k}\left(A_{k}\right)+\underset{A_{k}\subseteq \Omega ,\cap A_{k}\neq \phi }{\text{∑}}\left\{\left(1-\frac{K\times \left|\cap_{k=1}^{K}A_{k}\right|}{\sum_{k=1}^{K}\left|A_{k}\right|}\right)\prod_{k=1}^{K}m_{k}\left(A_{k}\right)\right\}\\ & =\underset{A_{k}\subseteq \Omega ,\cap A_{k}=\phi }{\text{∑}}\prod_{k=1}^{K}m_{k}\left(A_{k}\right)+\underset{A_{k}\subseteq \Omega ,\cap A_{k}\neq \phi }{\text{∑}}\prod_{k=1}^{K}m_{k}\left(A_{k}\right)-\underset{A_{k}\subseteq \Omega ,\cap A_{k}\neq \phi }{\text{∑}}\left\{\frac{K\times \left|\cap_{k=1}^{K}A_{k}\right|}{\sum_{k=1}^{K}\left|A_{k}\right|}\prod_{k=1}^{K}m_{k}\left(A_{k}\right)\right\}\\ & =1-\underset{A_{k}\subseteq \Omega ,\cap A_{k}\neq \phi }{\text{∑}}\left\{\frac{K\times \left|\cap_{k=1}^{K}A_{k}\right|}{\sum_{k=1}^{K}\left|A_{k}\right|}\prod_{k=1}^{K}m_{k}\left(A_{k}\right)\right\}\leq 1 \end{array}$$

If and only if $\cap_{k=1}^{K}A_{k}=\phi (k=1,\cdots ,k)$, the equal sign. Therefore, inequality 0 ≤ *D*_1…_*_K_* ≤ 1 is established. From the simple calculation, we can know that the form *D*_1…_*_K_* overcome the problem of *C*_1…_*_K_*.

#### 3.3.2. Improvement of $\text{difBet}P_{m_{1}}^{m_{2}}$

The problem of $\text{difBet}P_{m_{1}}^{m_{2}}$ is that $\text{difBet}P_{m_{1}}^{m_{2}}$ cannot measure the dissimilarity for more than three evidences. We put forward a new formula:

(12) $$\text{difBet}P_{1\cdots K}\underset{\begin{array}{c} 1\leq i,j\leq K\\ i\neq j \end{array}}{\max}\left\{\underset{A\subseteq \Omega }{\max}\left|\text{Bet}P_{i}\left(A\right)-\text{Bet}P_{j}\left(A\right)\right|\right\}$$

When *K* = 2, $\text{difBet}P_{12}=\text{difBet}P_{m_{1}}^{m_{2}}$, obviously *difBetP*_1…_*_k_* can measure the dissimilarity among more than two pieces of evidence simultaneously.

#### 3.3.3. Dualistic Exponential Function Form

We put forth the exponential dualistic function, through the association of multiple parameters, making up one-sidedness defect of a single parameter. In the same discernment frame Ω, with *K* (positive integer *K* ≥ 2) pieces of evidence, a dissimilarity measure expression as shown in Equation (13) is defined as BEF:

(13) $$BEF_{1\cdots K}=\frac{D_{1\cdots K}+\text{difBet}P_{1\cdots K}}{2}e^{-{\left(\frac{D_{1\cdots K}-\text{difBet}P_{1\cdots K}}{2}\right)}^{2}}$$

### 3.4. Comparison of Different Measure Methods

Comparing with the existing dissimilarity measurement methods, the advantages of our method can be shown in the following examples:

Example 1: Let three BBAs *m*_1_, *m*_2_, *m*_3_ be in the same discernment frame Ω = {*ω*_1_, *ω*_2_, *ω*_3_}:

$$\begin{array}{lll} m_{1}\left(\left\{\omega_{1}\right\}\right)=0.94, & m_{1}\left(\left\{\omega_{1},\omega_{2}\right\}\right)=0.03, & m_{1}\left(\left\{\omega_{1},\omega_{2},\omega_{3}\right\}\right)=0.03\\ m_{2}\left(\left\{\omega_{1}\right\}\right)=0.03, & m_{2}\left(\left\{\omega_{1},\omega_{2}\right\}\right)=0.03, & m_{2}\left(\left\{\omega_{1},\omega_{2},\omega_{3}\right\}\right)=0.94\\ m_{3}\left(\left\{\omega_{1}\right\}\right)=0.98, & m_{3}\left(\left\{\omega_{1},\omega_{2}\right\}\right)=0.01, & m_{3}\left(\left\{\omega_{1},\omega_{2},\omega_{3}\right\}\right)=0.01 \end{array}$$

The contrast results of different dissimilarity measurement methods are shown in Table 3.

From Table 3, we can see that *C*_12_ = *C*_13_ = 0 has been obtained by Shafer's dissimilarity measurement, which is not fit for people's intuition. In addition, other dissimilarity measurement methods can determine that the dissimilarity between *m*_1_ and *m*_3_ is far less than that between *m*_1_ and *m*_2_, which is much closer to human logic than Shafer's opinion. The two BBAs have visible differences about the dissimilarity measurement between *m*_1_ and *m*_2_; in terms of common sense, the dissimilarity between *m*_1_ and *m*_2_ would not be too large or too small. From the above several measurement methods, our method has more advantages obviously.

Example 2: Let three pair BBAs be in the same discernment frame Ω = {*ω*_1_, *ω*_2_, *ω*_3_, *ω*_4_, *ω*_5_}:

First pair:
  $$\begin{array}{c} m_{1}^{1}\left(\left\{\omega_{1},\omega_{2}\right\}\right)=0.9,m_{1}^{1}\left(\left\{\omega_{3}\right\}\right)=0.05,m_{1}^{1}\left(\left\{\omega_{4}\right\}\right)=0.05\\ m_{2}^{1}\left(\left\{\omega_{1},\omega_{2}\right\}\right)=0.05,m_{2}^{1}\left(\left\{\omega_{3}\right\}\right)=0.05,m_{2}^{1}\left(\left\{\omega_{4}\right\}\right)=0.9 \end{array}$$
Second pair:
  $$\begin{array}{l} m_{1}^{2}\left(\left\{\omega_{1},\omega_{2},\omega_{4}\right\}\right)=0.9,m_{1}^{2}\left(\left\{\omega_{3}\right\}\right)=0.05,m_{1}^{2}\left(\left\{\omega_{4}\right\}\right)=0.05\\ m_{2}^{2}\left(\left\{\omega_{1},\omega_{2}\right\}\right)=0.05,m_{2}^{2}\left(\left\{\omega_{3}\right\}\right)=0.05,m_{2}^{2}\left(\left\{\omega_{4}\right\}\right)=0.9 \end{array}$$
Third pair:
  $$\begin{array}{l} m_{1}^{3}\left(\left\{\omega_{1}\right\}\right)=0.9,m_{1}^{3}\left(\left\{\omega_{2},\omega_{3},\omega_{4},\omega_{5}\right\}\right)=0.1\\ m_{2}^{3}\left(\Omega \right)=1 \end{array}$$

From Table 4, we can see that there are more differences between the first pair and the third pair. Jousselme's method and Jia's method cannot separate these two kinds of circumstances. Liu's method and Guo's method reflect the different between the first pair and the third pair. It is difficult to pick out the threshold in Liu's method, which lacks operability. For example, we cannot quantitatively distinguish the conflict between <0, 0.7> and <0.0975, 0.55> quantitatively. The dissimilarity value of Guo's method changes greatly from the first pair to the third pair while our method reflects the relatively stable property gradually from the first pair to the third pair gradually.

Example 3: Let Ω be a discernment frame with 20 elements. We use 1, 2, *etc*. to denote element 1, element 2 in the discernment frame. The first BBA *m*_1_ is defined as:

$$m_{1}\left(\left\{2,3,4\right\}\right)=0.05,\;m_{1}\left(\left\{7\right\}\right)=0.05,\;m_{1}\left(\Omega \right)=0.1,\;m_{1}\left(A\right)=0.8$$

where *A* is a subset of Ω. The second BBA is *m*_2_({1,2,3,4,5}) = 1.

**Figure 1.**
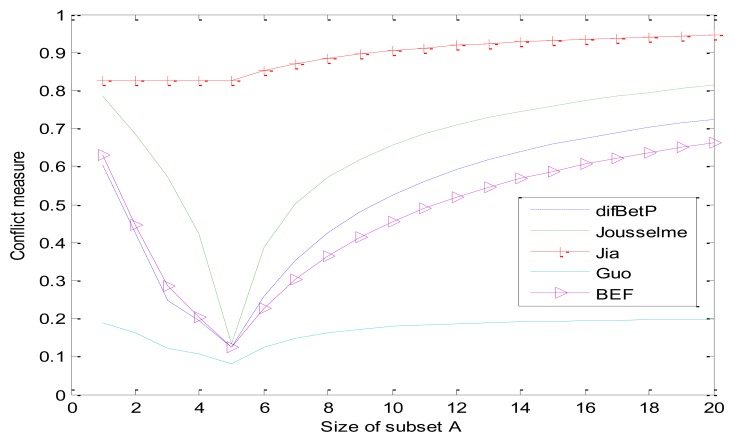
Comparison of different methods when subset *A* changes.

There are 20 cases where subset *A* increases one element at a time, starting from case 1 with *A* = {1} and ending with case 20 with *A* = Ω as shown in Table 5. The comparisons of different dissimilarity measure methods for these 20 cases are detailed in Table 5 and graphically illustrated in Figure 1. As can be seen from Table 5, value *C*_12_ always equals to 0.05 whether the size of subset *A* changes or not, which means that it cannot reasonably reflect the conflict degree between evidences. The results also indicate that all five dissimilarity measures change along with the size of *A*. When *A* = {1,2,3,4,5}, all values reach the minimum. The curves of Guo's and Jia's method are extreme cases. The value of Jia's method is worth so much more than Guo's. According to the appraisement criterion in [24], our method (BEF) is close to the curve of *difBetP* and reasonable.

All in all, our improved dissimilarity measure method has three advantages. Firstly, it is much closer to human logic and has no one ticket veto problem. Secondly, it overcomes the operational problem of existing dualistic conflict measure methods. Thirdly, it can measure the conflict among any pieces of evidence simultaneously and face interchangeability and combinability.

## 4. Evaluation Method of the Discounting Factor

In this section, the static discounting factor is assessed from a training set by comparing the sensor reading with the truth, which is based on the study of last section. Our method permits us to assess the static discounting factors of individual sensors. Different from the static method, the dynamic discounting factor is assessed in the process of target recognition, which is always on the test set. We deem that the sensor whose evidence in accordance with those of majority sensors is reliable comparatively. Then we bring forward an evaluation method of dynamic reliability based upon our improved dissimilarity measure. Different methods are discussed in the end of this section.

### 4.1. Problem Description

The static discounting factor of sensor *S_k_* (*k* ∈ {1,…, *K*}) is assumed to be evaluated. Let Г = {*o*_1_,…,*o_n_*} denote the training set of *n* targets and Ω = {*ω*_1_, *ω*_2_,…, *ω_p_*} denote the set of *p* classes.

The sensor reading about the class of each target *o_j_* o Γ is represented by a BBA on the set Ω. In a general training set, the class of each target is certain. While the knowledge of the truth often comes from uncertainty, risk, and ignorance [2] in realistic problems, and it can be represented by the belief function theory. In another word, the sensor reading and truth value can be represented by BBAs so that we can design the unified model.

In order to investigate the recognition performance of a fusion system across-the-board, the evaluation of dynamic reliability of each sensor is an important issue. When the real-time observation environment changes relative to the training environment, such as the decline and the invalidation of the sensor performance caused by environment noise and hostile interference, the static reliability and discount factor from the preliminary training no longer reflect the sensor performance and current status independently. Therefore, static evaluation of the sensor reliability is not enough, and the reliability of each sensor must be estimated dynamically in the fusion system.

### 4.2. The Existing Methods of Evaluating the Discounting Factor

Elouedi [6] has developed a method for assessing the sensor reliability in classification problems, while the pignistic transformation leads to the loss of information and bring about an increase of uncertainty.

Guo [2] has calculated the static and dynamic discounting factor based on the Jousselme distance measure, which has some defects in fact. Likewise, the static discounting factor can only distinguish different recognition performance between sensors on the overall, but cannot handle different target categories.

The proposed method of Yang [8] obtains the reliability factor of current identification evidence based on the sensor confusion matrix of *a priori* static information and its current output decision of dynamic information. However, this method has lost some original information.

Elouedi's method [9], which is simpler than Yang's, has put forward the idea of regarding the average correct classification rate as static reliability factors. Hence, the same problem of Yang's method also exists in Elouedi's method.

Xu' method [12] constructs a dissimilarity matrix whose elements are obtained by applying the cosine similarity measure method to evidence similarity in pignistic vectors. Obviously, the pignistic transformation has the defect of costing the loss of dynamic information.

### 4.3. Our Evaluation Method of Static Discounting Factor

Static reliability evaluation of sensors is a process of obtaining static discounting factors based on the training set. It is actually to obtain prior knowledge and performance of each sensor, and thus quantify sensor static reliability better. There are several key problems which must be considered: reasonable evidence dissimilarity measure under different conditions; the reliability evaluation of sensor which is based on the different categories of output; how to make full use of information on limited training samples.

Guo's method is based on the Jousselme distance measure. The distance metric may not conform to the common sense, as shown in Example 1. We adopt the method of last section based on the improved measure BEF (*m*_1_, *m*_2_).

Next, we start with the reliability evaluation based on the output of maximal pignistic probability. Based on the acquisition method of static discounting factor of Guo's [2] and Elouedi's *T_f_* method [9], we can only distinguish among different sensors in the overall recognition, and cannot indicate the sensor's ability in different target categories. Actually, recognition ability in different categories of sensors is usually diverse. Therefore, we should estimate the recognition reliability on different categories for each sensor, we can quantify the reliability of each sensor more accurately in this way. The static reliability evaluation based on the output of maximal pignistic probability has been adopted then.

For each training target *o_j_* ∈ Γ, let the BBA *m*{*o_j_*}[*S_k_*] denote the reading of sensor *S_k_* aiming to target *o_j_* and the BBA *m*{*o_j_*} denote *o_j_*. Let *BetR*{*o_j_*} represent the pignistic probability and *ω_t_* denote the corresponding element of the maximum pignistic probability about target *o_j_*, then:

(14) $$t=\arg\left\{\underset{i}{\max}\left\{\text{BetP}\left\{o_{j}\right\}\left(\omega_{i}\right)\right\}\right\}$$

We can obtain the static discounting factor $a_{k,t}^{s}(o_{j})$ of target *o_j_*:

(15) $$\alpha_{k,t}^{s}\left(o_{j}\right)=\alpha_{k}^{s}\left(o_{j}|\omega_{t}\right)=\text{BEF}\left(m_{k}\left[o_{j}|\omega_{t}\right],m_{0}\left[o_{j}\right]\right)$$

The factor $a_{k,t}^{s}(\left(o_{j}|\omega_{t}\right))$ denotes the static discounting factor under the condition of decision for being *ω_t_*, where *s* denotes “the static”, *k* denotes the serial number of sensor, *t* denotes the label of the class, in Equations (14) and (15), we regard the maximum pignistic probability as a prerequisite condition, which is the decision base and an important thought of TBM. We still adopt the values of *m*(•) to calculate the discounting, so the process is actually without information loss.

As known in Equation (15), the static discounting factor of each sensor is related to the dissimilarity measure between its reading and actual value.

In the training set Γ = {*o*_1_,…,*o_n_*} of *n* targets, we define *n_l_* (*l* = 1,2,…,*p*) as the number of the making decision equal to *ω_l_*. For each target in the set Γ, by repeating Equation (15), we get *n* static discounting factors of containing *p* classes, denoting as follows:

$$\begin{array}{cc} \alpha_{k,l}^{s}\left(o_{q_{1}}\right),\ldots \alpha_{k,l}^{s}\left(o_{q_{n_{l}}}\right) & l=1,2,\ldots ,p \end{array}$$

Naturally, we get the static discounting factor under the condition of each class via simple averaging operation:

(16) $$\begin{array}{cc} \alpha_{k,l}^{s}=\frac{1}{n_{l}}\overset{q_{n_{l}}}{\underset{q=q_{1}}{\text{∑}}}\alpha_{k,l}^{s}\left(o_{q}\right) & l=1,2,\ldots ,p \end{array}$$

Furthermore, we acquire the static reliability factor of the p-dimensional vector as follows:

(17) $$\alpha_{k}^{s}=\left(\alpha_{k,1}^{s},\alpha_{k,2}^{s},\ldots ,\alpha_{k,p}^{s}\right)$$

### 4.4. Our Evaluation Method of Dynamic Discounting Factor

The paper has an essential premise: each sensor is independent. In this premise, we follow the idea of Guo's method [2] and believe that the sensor in accord with the output evidences of most sensors is relatively reliable. We calculate the inconsistency by our improved dissimilarity measure method.

Suppose the total number of sensors is *K* and the conflict between the BBAs of different reliability sensors has been found out, according to the explanation in [7], which means that there is at least an unreliable sensor. When the number of sensors is large, these sensors are more reliable and other sensors against them are not, if the output evidences from most of sensors have enormous support on one category or a few categories. Then, as described in [2], the dynamic reliability evaluation should be achieved on the basis of the opinion for majority sensors, the dynamic reliability and discount factor should be the consistency embodiment between the output of each sensor and the majority opinion.

In order to measure the consistency between the output of each sensor *S_k_* (*k* ∈ {1,…, *K*}) and the majority opinion, while we should construct the majority opinion first. To the BBA *m_k_* (*k* ∈ {1,…, *K*}) of *K* sensors output, this paper regards the mean value of BBAs *m̅* as a characterization of the majority opinion. To ∀*x* ⊆ *Ω*, *m̅* is calculated as follows:

(18) $$\bar{m}\left(A\right)=\sum_{k=1}^{K}m_{k}\left(A\right)/K$$

The foundation of constructing a majority opinion is the set supported by most of *m_k_* and it will also obtain more support in *m̅*; on the other hand, the corresponding set acquires less support degree in *m̅*.

The dissimilarity measure *BEF*(*m_k_*[*o*], *m̅*[*o*]) between each *m_k_* and *m̅* can be calculated by Equation (13). Larger *BEF*(*m_k_*[*o*], *m̅*[*o*]) means that the output BBA *m_k_* of sensor *S_k_* is more inconsistent with the majority opinion, and its reliability is lower, thus, the discounting factor should be higher. On the other hand, if the output BBA *m_k_* of sensor *S_k_* is more consistent with the majority opinion, then its reliability is higher, and the discounting factor should be lower.

Therefore, *BEF*(*m_k_*[*o*], *m̅*[*o*]) can be used as the dynamic discount factor of sensor *S_k_*:

$${\alpha \prime }_{k}^{d}=\text{BEF}\left(m_{k}\left[o\right],\bar{m}\left[o\right]\right)$$

and:

$${R\prime }_{k}^{d}\left(s_{k}\right)=1-\text{BEF}\left(m_{k}\left[o\right],\bar{m}\left[o\right]\right)$$

which is the relative dynamic reliability factor of sensor *S_k_*. The superscript *d* denotes the dynamic reliability and discount factor. We can also obtain absolute reliability of the sensor *S_k_*:

(21) $$R_{k}^{d}\left(sk\right)=\frac{{R\prime }_{k}^{d}\left(s_{k}\right)}{\underset{k=1,\cdots ,K}{\max}\left\{{R\prime }_{k}^{d}\left(s_{k}\right)\right\}}$$

### 4.5. Analysis of Several Evaluation Methods

Compared with other methods, the advantages of our evaluation methods are:

(1). We can calculate the dissimilarity measure between BBAs directly, which are derived from the reading of the sensor and actual value of data. The output category based on maximum pignistic probability is regarded as conditions of fine discounting, which can use the samples fully and there is no additional loss in the calculation process.
(2). The dissimilarity measure and mean operation can correspond to either certain training samples or general training samples, and also have considerable flexibility.
(3). Compared with other methods, our method has no information loss, the computational complexity of our method is fairly lower, and the reliability evaluation would be more reasonable and accurate.

## 5. The Adaptive Combination Method of Static and Dynamic Discounting Factor

In this section, we will study the combination of static and dynamic discounting factor penetratingly. Based on [2], this paper has proposed an adaptive combination method of static and dynamic discounting factor, and this method can adapt to environmental changes such as noise and false target, which provides a new thought of the combination for the static and dynamic discounting.

### 5.1. Problem Description

**Figure 2.**
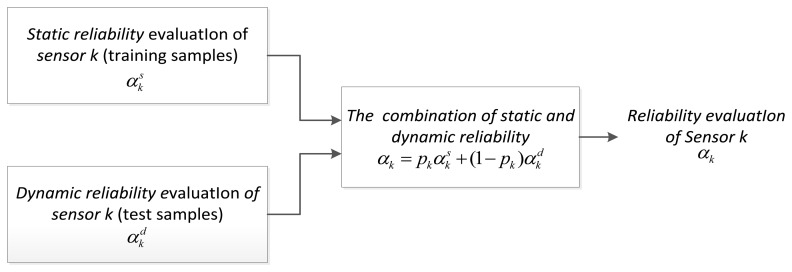
Implementation framework of Guo's combining method.

Guo [2] has argued that the discounting factor from static evaluation can be regarded as a performance indicator of sensors, or the prior knowledge for subsequent application. When the sensor is used in different situations or at different stages, its reliability will change. Dynamic discounting factor has obtained by real-time training reflects the current performance and background information of the sensor. In normal conditions, *a priori* (or static) reliability of a dynamic environment is very important, and it should be combined with the fusion process. Guo [2] has put forward a combination method for two kinds of discounting factors, and chose a combination method which used weighted average to get the comprehensive discounting factor:

$$\alpha_{k}=p_{k}\alpha_{k}^{s}+(1-p_{k})\alpha_{k}^{d}$$

where *p_k_* ∈ [0,1] is a static weight. In particular, if the current actual environment is quite different from the static learning condition, the sensor is sensitive to the change of the background, then taking *p_k_* ≤0.5 is appropriate. On the contrary, if the actual environment is similar with static evaluation condition, we should assume *p_k_* ≥0.5. The implementation process of Guo's combining method is shown in Figure 2.

While in this method, the static weight *p_k_* is determined beforehand by empiric completely, and cannot really change along with the actual environment. When the environmental condition has a big change, the sensor may be affected by a lot of factors, which eventually leads to the sharp decline of fusion system performance. Therefore, we must develop a new combination method which is adaptive to the environment.

### 5.2. Our Adaptive Combining Method

**Figure 3.**
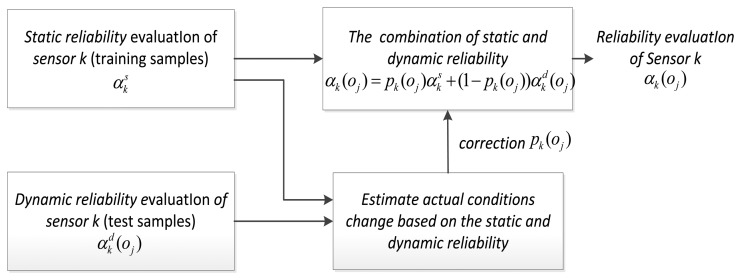
Implementation framework of the our combining method.

To achieve adaptive combining of static and dynamic discounting factor, we use a new implementation framework, showing in Figure 3. The expression is:

(23) $$\alpha_{k}(o_{j})=p_{k}(o_{j})\alpha_{k}^{s}+(1-p_{k}(o_{j}))\alpha_{k}^{d}(o_{j}),\;j=1,\ldots ,N$$

*N* is the number of test samples; *p_k_*(*o_j_*) is a static variable changing along with the target classification or recognition process. When the background environment changes, the value alters with the change of the sensor performance, which leads to the real-time change of the sensor performance; hence, the core of the problem is the estimate of static weight.

In order to simplify the problem, this paper makes the following assumptions: in the whole sensor group, the performance of single sensor or several sensors will be affected by the environment, and the performance of most of the other sensors remains the same, which is more common and reasonable in the heterogeneous sensors.

In accordance with the above assumptions, we come up with the following ideas. First of all, from the training samples, we obtain the sequence of dynamic discounting factor for each sensor, and then use the nonparametric estimation method to acquire the probability density function of dynamic discounting variables. In the testing process, according to the real-time dynamic discounting of each target, we can calculate the matching degree between the current sensor performance and static environment sensor performance and catch hold of the sensor performance. This paper has arranged two steps to realize the adaptive combination of static and dynamic discounting factors.

#### 5.2.1. Based on the Training Samples, the Parzen-Window Estimate Method is Used to Obtain Dynamic Probability Density Function of Discounting Variables

Question: given a sequence of independent and identically distributed random variables *x*_1_, *x*_2_,…, *x_n_* with common probability density function *f*(*x*), how can *f*(*x*) be estimated? In practical problems, in the sequence values of sensor dynamic discounting often do not know the overall distribution form, and the function of some parameters cannot be constructed. Therefore, this paper uses the Parzen-window estimate probability density function of dynamic discounting based on the training samples.

The Parzen-window estimate method [27,28] is an effective nonparametric estimation method which is able to take advantage of the known samples to estimate the overall distribution of density function. The basic idea is using the mean value of each point within a certain range of density to estimate the overall density function. The specific method is:

Assuming *x* is any point in the n-dimensional space, in order to estimate distribution probability density *p*(*x*) of *x*, we form a hypercube whose center is *x* and side length is *h_n_*, so its volume is $V_{n}=h_{n}^{d}$ and *n* is the number of total samples.

In order to calculate the number *n_V_n__* of samples which fall into the volume *V_n_*, we construct a function:

(24) $$\varphi ({\left[u_{1},u_{2},\cdots ,u_{d}\right]}^{T})=\{\begin{array}{ll} 1, & if|u_{j}|\leq ½,\;j=1,2,\cdots ,d\\ 0, & \text{other} \end{array}$$

Now, the number of samples with *V_n_* is $n_{V_{n}}=\sum_{i=1}^{n}\phi \left(\frac{x-x_{i}}{h_{n}}\right)$, in the point *x*, the estimation value of the probability density *p*(*x*) is:

(25) $${\hat{p}}_{n}(x)=\frac{1}{n}\sum_{i=1}^{n}\frac{1}{V_{n}}\varphi (\frac{x-x_{i}}{h_{n}})$$

Equation (25) is the basic formula of the estimation method of Parzen window (*V_n_* uses in the formula, of course, is not necessarily limited to the cube, and can also be a more general form), and define the kernel function (or the window function):

$$K(x,x_{i})=\frac{1}{V_{n}}\varphi (\frac{x-x_{i}}{h_{n}})$$

and *K*(*x*, *x_i_*) satisfies two conditions: (1) *K*(*x*, *x_i_*) ≥ 0; (2) ∫*K*(*x*, *x_i_*)*dx* = 1.

Common window function has a variety of forms. The window function and normal window function are most widely used, and the specific form is:

(a) The window function:
  (26) $$K(x,x_{i})=\{\begin{array}{ll} 1, & if\left|x^{j}-x_{i}^{j}\right|\leq ½,\;j=1,2,\cdots ,d\\ 0, & \text{other}. \end{array}$$
(b) The normal window function:
  (27) $$K(x,x_{i})=\frac{1}{\sqrt{2\pi }}\exp\left\{-\frac{1}{2}{\left(x-x_{i}\right)}^{2}\right\}.$$

**Figure 4.**
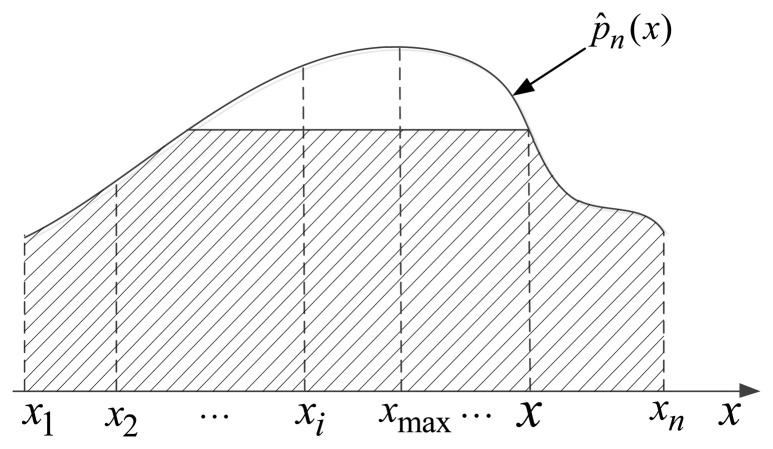
The estimation of overall probability density function *p̂_n_(x)*.

Note that, in the basic formula for estimation method of the Parzen-window, window width *h_n_* is a quite important parameter. When the number of samples is limited, *h_n_* has a major influence on the effect of estimation. In practical calculations, we adopt the normal window function and set $h_{n}=1/\sqrt{n}$. Without loss of generality, we get the estimation of overall probability density function *p̂_n_(x)* which is shown in Figure 4.

For *n* targets in the training samples, we denote the dynamic discounting factor of the sensor as $\left(a_{k}^{d}(o_{1}),a_{k}^{d}(o_{2}),\cdots ,a_{k}^{d}(o_{n})\right)$. In Equation (25), let $v_{n}=h_{n}=1/\sqrt{n}$, $\text{xi}=a_{k}^{d}(o_{i})$, then the probability density function of test target *o* is:

(28) $${\hat{p}}_{n}(\alpha_{k}^{d}(o))=\frac{1}{n}\sum_{i=1}^{n}\sqrt{n}\cdot \varphi (\frac{\alpha_{k}^{d}(o)-\alpha_{k}^{d}(o_{i})}{1/\sqrt{n}})$$

Let ${\hat{p}}_{n}(a_{k}^{d}(o_{\text{max}}))=\begin{array}{c} \max\\ i \end{array}({\hat{p}}_{n}a_{k}^{d}(o_{\text{max}})))$, for test target *o*, if ${\hat{p}}_{n}(a_{k}^{d}(o))$ is much closer to the maximum value ${\hat{p}}_{n}(a_{k}^{d}(o_{ma}))$, then it represents that this sensor is much closer to the performance of static environment. Thus, the environment has no change or effect on the current sensor. On the contrary, the current sensor performance varies greatly, if ${\hat{p}}_{n}(a_{k}^{d}(o))$ is far more away from the maximum value ${\hat{p}}_{n}(a_{k}^{d}(o_{ma}))$, which shows that the current performance of sensors has a big gap with the static performance. Hence, we use a ratio of the area marked by the oblique line in Figure 4 and the total area under the probability density function to represent the matching degree for current performance of sensor and the static performance naturally:

(29) $$C(\alpha_{k}^{d}(o))=\frac{\overset{n}{\underset{i=1,{\hat{p}}_{n}(\alpha_{k}^{d}(o_{i}))\leq {\hat{p}}_{n}(\alpha_{k}^{d}(o))}{\text{∑}}}{\hat{p}}_{n}(\alpha_{k}^{d}(o_{i}))}{\sum_{i=1}^{n}{\hat{p}}_{n}(\alpha_{k}^{d}(o_{i}))}$$

#### 5.2.2. Dynamic Learning of Discounting Weights Based on Fuzzy Set Theory

**Figure 5.**
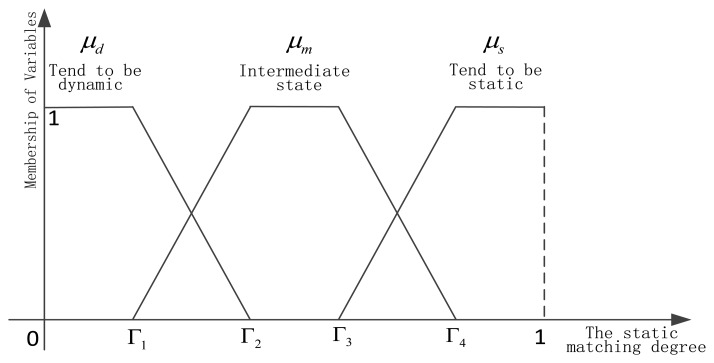
Relational graph of fuzzy variables and static matching degree.

After obtaining the matching degree of current performance and the static performance based on each sensor, we need to realize dynamic learning of static and dynamic discounting weights. Because the matching degree can reflect that the current sensor is in a static environment, or away from the static environment, or in the intermediate state, which has a certain ambiguity, so the fuzzy set theory [29] has been used. The integration researches of belief function theory and fuzzy set theory can be found in [30–34]. In this paper, the matching degree is decomposed into three fuzzy variables, as shown in Figure 5.

Dynamic fuzzy variable *μ_d_*, intermediate state fuzzy variable *μ_m_*, and static fuzzy variable are listed below respectively:

(30) $$\mu_{d}=\{\begin{array}{cc} 1 & if\;0\leq C(\alpha_{k}^{d}(o))<\Gamma_{1}\\ \frac{\Gamma_{2}-C(\alpha_{k}^{d}(o))}{\Gamma_{2}-\Gamma_{1}} & if\;\Gamma_{1}\leq C(\alpha_{k}^{d}(o))<\Gamma_{2}\\ 0 & \text{else} \end{array}$$

(31) $$\mu_{m}=\{\begin{array}{cc} \frac{C(\alpha_{k}^{d}(o))-\Gamma_{1}}{\Gamma_{2}-\Gamma_{1}} & if\;\Gamma_{1}\leq C(\alpha_{k}^{d}(o))<\Gamma_{2}\\ 1 & if\;\Gamma_{2}\leq C(\alpha_{k}^{d}(o))<\Gamma_{3}\\ \frac{\Gamma_{4}-C(\alpha_{k}^{d}(o))}{\Gamma_{4}-\Gamma_{3}} & if\;\Gamma_{3}\leq C(\alpha_{k}^{d}(o))<\Gamma_{4}\\ 0 & \text{else} \end{array}$$

(32) $$\mu_{s}=\{\begin{array}{cc} 1 & if\;\Gamma_{4}\leq C(\alpha_{k}^{d}(o))<1\\ \frac{C(\alpha_{k}^{d}(o))-\Gamma_{3}}{\Gamma_{4}-\Gamma_{3}} & if\;\Gamma_{3}\leq C(\alpha_{k}^{d}(o))<\Gamma_{4}\\ 0 & \text{else} \end{array}$$

Let the numbers of training samples and test samples be *n* and *N* respectively, we design the adaptive dynamic and static combination method:

(33) $$\begin{array}{ll} \alpha_{k}(o_{j}) & =\frac{n+\sum_{l=1}^{j}\left(\mu_{s}+0.5\mu_{m}\right)}{n+\sum_{l=1}^{j}\left(\mu_{s}+\mu_{m}+\mu_{d}\right)}\alpha_{k}^{s}\\ & +\frac{\sum_{l=1}^{j}\left(\mu_{d}+0.5\mu_{m}\right)}{n+\sum_{l=1}^{j}\left(\mu_{s}+\mu_{m}+\mu_{d}\right)}\alpha_{k}^{d}(o_{j}), \end{array}\;j=1,\ldots N$$

The weight of the static discounting in Equation (23) is computed by Equation (33) as follows:

(34) $$p_{k}(o_{j})=\frac{n+\sum_{l=1}^{j}\left(\mu_{s}+0.5\mu_{m}\right)}{n+\sum_{l=1}^{j}\left(\mu_{s}+\mu_{m}+\mu_{d}\right)}$$

Before the test, the dynamic and static weights are 0 and 1 respectively, which is logical. Because there is no dynamic information before that. When the test goes on, we can match the current performance of the sensor and static performance. Then from three fuzzy variables, we accomplish the self-learning to environment interference and other unknown factors. The intermediate state variable *μ_m_* is an important factor for improving system robustness. After two steps, this paper builds the basic framework of adaptive combination of dynamic and static sensor reliability evaluation.

## 6. Experimental Results and Discussion

We perform three series of experiments. The performances of various static methods are firstly compared to those of classifiers trained using the U.C.I datasets. Then we research the behavior of several static methods. In the third series of experiments, we can obtain the related conclusions by comparing two combination methods of static and dynamic discounting in different situations. The experimental setup is described in Section 6.1, and the results are presented and discussed in Sections 6.2–6.4.

### 6.1. Experimental Setup

#### 6.1.1. Experimental Datasets

The datasets used in these experiments are summarized in Table 6. All targets in the dataset are divided into three equal parts. In fact, datasets for generating BBAs reflect the performance of the classifier.

#### 6.1.2. Construction of Basic Belief Assignment

In a single dataset, all the features are divided into three groups, each group contains several features and each group is the basis of classifiers. The description of generating classifiers is shown in Table 7.

To fairly compare the various methods, we adopt the construction method of [36] uniformly. The degree of support is defined as a function of the distance between two vectors, and the evidence of the *k* nearest neighbors is then pooled by using the Dempster's rule of combination. The main parameters (*k*, *α_o_*, *γ_q_*, *β* ([36], p. 809)) of generating BBAs are fixed in order to compare different methods. Let *α_o_* = 0.95 and *γ_q_* be determined separately for each class as $1/d_{q}^{\beta }$, where *d_q_* is the mean distance between two training vectors belonging to class *ω_q_*. The choice of *k* lists in Table 7 and *β* = 1 is adopted in our experiment.

### 6.2. Comparison of Methods for Evaluating the Static Discounting Factor

#### 6.2.1. Methodology

Let B be a dataset composed of *L* vectors (objects). Results obtained from different classifiers are given as follows:

(1). All targets in the dataset B are divided into three groups: the dataset for generating BBAs *B_BBAs_*, the training dataset *B_train_*, and the test dataset *B_test_*.
(2). Based on the dataset *B_BBAs_*, we can generate three classifiers through the above method [36] of generating BBAs. Every object in *B_test_* is used to evaluate the performances of three classifiers, whose classification results can be obtained by using the maximum pignistic probability rule. (*B_train_* is not used in this case)
(3). In order to make a decision, decisions obtained by three classifiers are then combined according to the majority vote method and the Dempster rule of combination method respectively.

The correct classification rates of classifiers and two methods are respectively shown in Table 8.

#### 6.2.2. Implementation

To implement different methods of evaluating the static discounting factor, the following steps are carried out:

(1). By testing every classifier based on the dataset *B_train_*, we can use different confusion matrices for different classifiers, which will be used by some evaluation methods. Then the static discounting factors of different methods are computed.
(2). For each object, based on the different static discounting factors and Equation (6), different BBAs are calculated by every classifier in the test set *B_test_*.
(3). Once the BBAs are obtained, the final results by the Dempster rule of combination can be computed according the formula: $m_{1⊕\cdots ⊕K}={⊕}_{i=1}^{K}m_{k}=m_{1}⊕\cdots ⊕m_{k.}$.
(4). Thus the final object can be classified by the maximum pignistic probability rule.
(5). Repeat step (2) until all the data in the test set are tested.

We compare the performances of various methods by evaluating the static discounting factors. The classification results via five kinds of methods are shown in Table 9. We can see that, the accuracy rates of fusion methods are higher than those of three classifiers. Apparently, our static method can get better results than others, which proves that our method is effective.

### 6.3. Comparison of Methods of Evaluating the Dynamic Discounting Factor

We have three classifiers based on the dataset *B_BBAs_*. To implement different methods of evaluating the dynamic discounting factor, the following steps should be carried out (*B_train_* won't be used in this case):

(1). For every object, different BBAs are calculated by every classifier in the test set *B_test_*, then the dynamic discounting factors of different methods are obtained.
(2). For the same object, based on the different dynamic discounting factors and Equation (6), different BBAs are calculated again by every classifier.
(3). Once the BBAs are obtained, the final results are gained by the Dempster rule of combination.
(4). Thus the final object can be classified by using the maximum pignistic probability rule.
(5). Repeat step (1) and (2) until all the data in the test set are tested.

We compare the performances of several methods using the dynamic discounting factors. As shown in Table 10, our dynamic method is better than others. As explained in Section 1, the dynamic reliability is calculated in the test process without using the training sets, so the results of the dynamic reliability methods are slightly poor than those of the static reliability methods.

### 6.4. Comparison of Methods of Combining the Static and Dynamic Discounting Factors

In order to compare the performances of different combination methods, a typical scenario has been designed, which is made up of a series of cases affected by the actual environment. A typical scenario is designed as follows. A reconnaissance ship with three heterogeneous sensors on the sea carries out the task of reconnaissance. This kind of scenario may include visual camera (VIS), infrared camera (IR) and Radar. There might be many unexpected conditions for reconnaissance ship on the sea, which affect the performance of the sensor. Based on this assumption, we design four experiments.

#### 6.4.1. A Fixed Environmental Interference Leading to the Performance Degradation of One Sensor

Since sea fog reduces visibility, the performance of the visual camera will decline. Then the dataset *B_BBAs_* is a reflection for the performance of the sensor. By adding a fixed uncertainty on output of one sensor, for example Gauss white noise, this situation is simulated very well.

**Figure 6.**
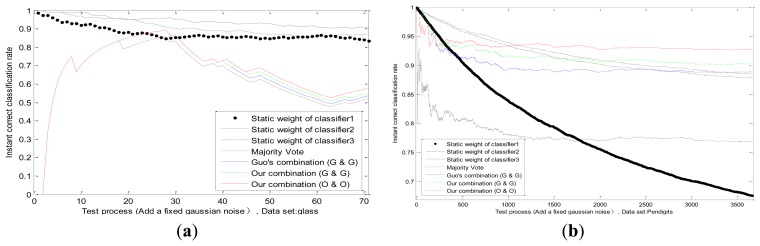
Instant result of different methods in the whole test process on dataset glass (a) and pendigits (b).

In our experiments we superimpose a fixed Gaussian white noise on the actual value of one classifier; whose standard deviation equals to two-thirds of actual value. The threshold in Equations (30, 31, 32) is Γ_1_ = 0.3, Γ_2_ = 0.4, Γ_3_ = 0.5, Γ_4_ = 0.6; and *n* is the number of training samples. We can observe the static weight changes of each classifier in the process of testing; and the correct classification rates of four methods: majority vote; Guo's combination [2] (Guo's static method and Guo's dynamic method; *p_k_* = 0.5); our combination (Guo's static method and Guo's dynamic method); and our combination (our static method and our dynamic method); which are shown in Figure 6 in different datasets. Firstly; we can see that the static weight of the first classifier decreases faster than other classifiers; which indicates that our combination method can detect the performance degradation on the first classifier. Secondly; in the combination of same methods (Guo's static method and Guo's dynamic method); our combination method is better than Guo's. In addition; our combination strategy has advantages on our methods than on Guo's methods; which verifies the effectiveness of our static and dynamic method.

**Figure 7.**
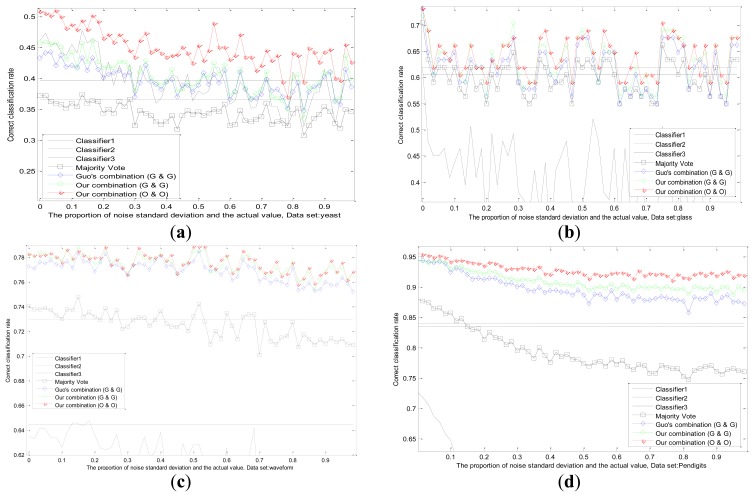
Results of different methods on dataset yeast (a), glass (b), waveform (c), and pendigits (d) by adding the fixed Gaussian noise.

We conduct experiments with the changes of datasets and values of standard deviation in Figure 7. The correct classification rates are compared by different methods when the fixed standard deviation changes. We design the changes of standard deviation for Gauss white noise as follows: steps for 60, step length for 1/60 of the actual values. Each standard deviation corresponds to a test result in different datasets as shown in Figure 7.

It can be observed that, the recognition ability of the first classifier drops sharply because of the influence of uncertainty, while the performances of other two classifiers remain the same. Experimental results in several datasets prove that, our adaptive combination method (our static method and our dynamic method) is the best, our adaptive combination method (Guo's static method and Guo's dynamic method) lists proxime accessit, and Guo's combination method (Guo's static method and Guo's dynamic method) is the third one. The results testify the finer capability of our static method, our dynamic method and our adaptive combination strategy.

#### 6.4.2. A Changing Environmental Interference Impacting the Performance Degradation of One Sensor

**Figure 8.**
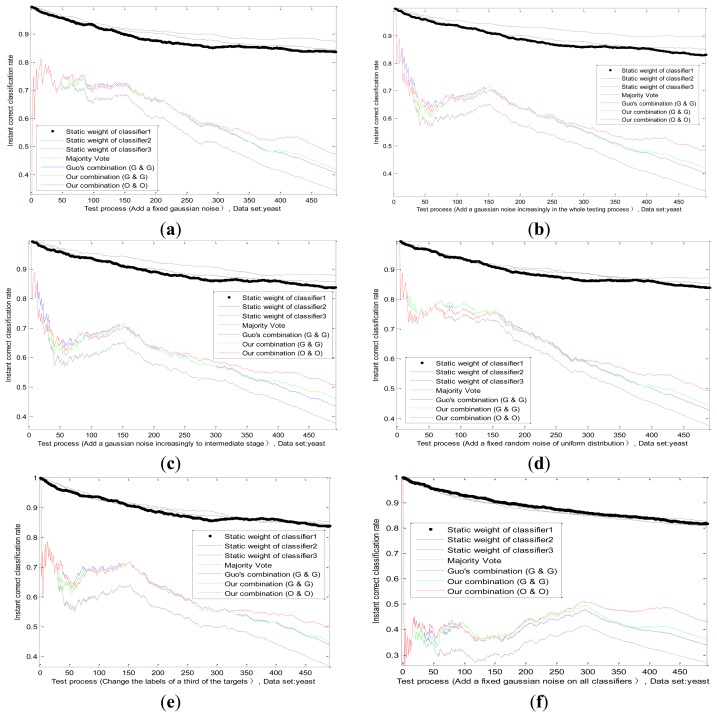
Instant results of different methods in the whole test process on different conditions. Add a fixed Gaussian noise on classifier 1 (a), Add a Gaussian noise on classifier 1 increasingly in the whole testing process (b), Add a Gaussian noise on classifier 1 increasingly to intermediate stage (c), Add a fixed random noise of uniform distribution on classifier 1 (d), Change partial labels of the targets of classifier 1 (e), Add a fixed Gaussian noise on all classifiers (f).

A fixed environmental interference has been discussed before. However, the actual situation is not static and marine environment is influenced by many factors. For example, the fog at sea may thicken or be thin, which will affect VIS. The detection performance of radar is directly related to the intensity of the sea clutter. Then we do an experiment on the dataset *B_BBAs_*, by adding a change uncertainty on actual value of one sensor, such as Gauss white noise or random noise, those situations are simulated. This paper designs four typical noises as follows. The effect of several methods is shown in Figure 8.

- Standard deviation of Gaussian noise adds fixedly in the whole testing process.(Figure 8a)
- Standard deviation of Gaussian noise adds increasingly in the whole testing process.(Figure 8b)
- Standard deviation of Gaussian noise adds increasingly until intermediate stage, and then the noise disappears in the remaining testing process. (Figure 8c)
- Noise model changes according to a random noise of uniform distribution. (Figure 8d)

We can see from Figure 8b, comparing with Figure 8a, that the static weight of the first classifier drops slower, but is still higher than other two classifiers, which reflects the increasing trend of the uncertainty. Before the middle stage, the static weight of the first classifier in Figure 8c declines in accordance with its static weight in Figure 8b, which is logical. After the middle stage, due to the disappearance of noise, the static weight of the first classifier has a rising trend, whose value is close to other classifiers. It certifies that the combination mechanism adjusts the weight according to the performance change of the sensor. Hence, we change the noise model and obtain the classification result in Figure 8d. There is no big difference between two noise models, which also proves the applicability of our method.

#### 6.4.3. The Enemy's False Goals Leading to the Wrong Recognition of One Sensor

When a reconnaissance ship detects enemy targets on the sea, sometimes the enemy may release some fake targets, which will lead to the completely wrong identification of some sensors on ships. For example, the perfect stealth technology of enemy targets can mislead the radar. In this situation, we study and analyze the effect of adaptive combination method for static and dynamic discounting factors. By changing some labels of actual target for one sensor in the dataset *B_BBAs_*, we can simulate this situation substantially. By modifying the labels of a third of the targets, the results of several methods are obtained in Figure 8e. We find that our adaptive combination mechanism can deal with the changes of sensor performance caused by the enemy false target.

#### 6.4.4. The Enemy's Intentional Interference Leading to the Performance Degradation of All Sensors

When enemy targets find that this reconnaissance ship is detected nearby, the enemy targets often cause the performance degradation of all sensors, through the omnibearing interference. Under this kind of situation, the effect of combining method proposed in this paper is studied by comparing with other methods. In our experiment, we superimpose a fixed Gaussian white noise on the actual value of all classifiers, whose standard deviation is equal to two-thirds of actual value. The result is shown in Figure 8f. The change trend of static weight of all classifiers is consistent basically, and this corresponds with the intuitive logic.

It is important to note that, classification results of the adaptive combination method are better than Guo's combination method and the majority vote method in the above cases, which show the effectiveness and applicability of our methods (our static method, our dynamic method and our adaptive combination strategy).

## 7. Conclusions

In this paper, a new sensor reliability algorithm is present to correct the basic belief assignment from each sensor, which can improve the recognition accuracy and robustness of the fusion system. This paper mainly has two innovative aspects:

First of all, an improved dissimilarity measure based on dualistic exponential function has been designed. This paper integrates the advantages of both BBM type and distance type dissimilarity measures. The improved measure method is more intuitive for people and overcomes the operational problem of the existing dualistic dissimilarity measure methods. On account of this point, we assess the static reliability from a training set by the local decision of each sensor and dissimilarity measure between evidences. Meanwhile, the dynamic reliability factors are gained from every test target by the dissimilarity measure, which is between the output information of each sensor and the consensus.

Secondly, based on the Parzen-window estimation and fuzzy theory, this paper introduces an adaptive method of combining static and dynamic discounting. The static weight of original method is determined beforehand by experts completely and cannot be changed with the actual environment. For solving this problem, we adopt the Parzen-window estimate to acquire the matching degree of the current performance for sensors and the static performance based on the training samples. Then, our implementation mechanism can be suitable for different kinds of target environment via three fuzzy variables, which shows the classification accuracy and the robustness of our methods by comparing with other methods.

We have used only the evaluation methods of sensor reliability on the classical discounting. In future, we will combine the adaptive method with different discounting mechanisms, which may improve the performance of fusion system further.

## Figures and Tables

**Table 1.** Analysis of different dissimilarity measure methods.

**Categories**	**Methods**	**Measure Expressions**	**Advantages**	**Disadvantages**
BBM	Shafer [1]	$C_{1\cdots K}=\underset{\cap_{k=1}^{K}X_{k}=\phi ,X_{k}\subseteq \Omega }{\text{∑}}\overset{K}{\underset{k=1}{\text{∏}}}m_{k}\left(X_{k}\right)$	It can measure the dissimilarity of more than three pieces of evidences; and the implement efficiency is high.	Its results are often counterintuitive, for example the problem of one-vote veto.

	Jia [13]	$\begin{array}{l} GC_{1\cdots K}=\underset{A_{k}\subseteq \Omega }{\text{∑}}\{\left(1-\frac{\left|\cap_{k=1}^{K}A_{k}\right|}{\prod_{k=1}^{K}\left|A_{k}\right|}\right)\prod_{k=1}^{K}m_{k}\left(A_{k}\right)\}\\ \left|\cdot \right|\text{denotes the cardinality} \end{array}$	It includes both direct dissimilarity and potential conflict.	In different evidence conditions, the dissimilarity measure results between evidences are large relatively.

Distance	Wang [21]	$R_{\text{BBA}}\left(m_{1},m_{2}\right)=\underset{A\subseteq \Omega }{\text{∑}}\frac{\left|m_{1}\left(A\right)-m_{2}\left(A\right)\right|}{2}$	Its form is intuitive and simple with high execution efficiency.	The measure is not careful without considering the compatible parts of focal elements.

	Jousselme [22]	$\begin{array}{l} d_{\text{BBA}}\left(m_{1},m_{2}\right)=\sqrt{\frac{1}{2}{\left(m_{1}-m_{2}\right)}^{T}D\left(m_{1}-m_{2}\right)}\\ \forall A,B\subseteq \Omega ,D\left(A,B\right)=\frac{\left|A\cap B\right|}{\left|A\cup B\right|},\left|\cdot \right|\text{denotes the cardinality} \end{array}$	It describes the dissimilarity between evidences and has the support of distance axiom.	Its computation is large when the number of elements for discernment framework is large, and it is not reasonable sometimes.

Complex	Liu [24]	$\begin{array}{l} cf\left(m_{1},m_{2}\right)=\langle C_{12},\text{difBet}P_{m_{1}}^{m_{2}}\rangle \\ \text{difBet}P_{m_{1}}^{m_{2}}=\underset{A\subseteq \Omega }{\max}\left|\text{Bet}P_{1}\left(A\right)-\text{Bet}P_{2}\left(A\right)\right| \end{array}$	It includes both BBM type and distance type of dissimilarity measures.	The dualistic dissimilarity measure leads to the complexity of determining the threshold, which has not uniform criterion.

	Guo [20]	$cf_{G}=\frac{C_{12}+\text{difBet}P_{m_{1}}^{m_{2}}}{2}e^{-\left|C_{12}-\text{difBet}P_{m_{1}}^{m_{2}}\right|}$	It overcomes the operation complexity of dualistic dissimilarity measure.	The results of the dissimilarity measure are illogical sometimes.

**Table 2.** Different consistency measure functions.

**Name**	**Sokal & Sneath**	**Dice**	**Ochiai**	**Fixsen & Mahler**
Function Form	$\frac{\left|A\cap B\right|}{2\left|A\cup B\right|-\left|A\cap B\right|}$	$\frac{2\left|A\cap B\right|}{\left|A\right|+\left|B\right|}$	$\frac{\left|A\cap B\right|}{\sqrt{\left|A\right|\left|B\right|}}$	$\frac{\left|A\cap B\right|}{\left|A\right|\left|B\right|}$

**Table 3.** Contrast results of different dissimilarity measurement methods.

**Evidence**	**Methods**				

	**<*C*_12_**, **difBetP> [24]**	**Jousselme [22]**	**Guo [20]**	**Jia [13]**	**BEF**
*m*_1_ and *m*_2_	<0, 0.6067>	0.7430	0.1654	0.6429	0.5293
*m*_1_ and *m*_3_	<0, 0.0233>	0.0283	0.0114	0.0460	0.0279

**Table 4.** Contrast of different dissimilarity measurement methods.

**Evidence**	**Methods**				

	**<*C*_12_**, **difBetP> [24]**	**Jousselme [22]**	**Guo [20]**	**Jia [13]**	**BEF**
The first pair	<0.9075,0.85>	0.85	0.8296	0.93	0.878
The second pair	<0.0975,0.55>	0.6946	0.2059	0.6675	0.5306
The third pair	<0,0.7>	0.8062	0.1738	0.8	0.6543

**Table 5.** Comparisons of different conflict measurement methods.

**Cases**	**<*C*_12_**, **difBetP> [24]**	**Jousselme [22]**	**Guo [20]**	**Jia [13]**	**BEF**
A = {1}	<0.05, 0.605>	0.78581	0.18801	0.825	0.63
A = {1,2}	<0.05, 0.42667>	0.68666	0.16353	0.825	0.4458
A = {1,2,3}	<0.05, 0.24833>	0.57053	0.12233	0.825	0.285
A = {1,…,4}	<0.05, 0.195>	0.42367	0.10597	0.825	0.2032
A = {1,…,5}	<0.05, 0.125>	0.13229	0.081178	0.825	0.1237
A = {1,…,6}	<0.05, 0.25833>	0.38837	0.12517	0.85167	0.2266
A = {1,…,7}	<0.05, 0.35357>	0.50292	0.14895	0.87071	0.304
A = {1,…,8}	<0.05, 0.425>	0.57053	0.16323	0.885	0.3648
A = {1,…,9}	<0.05, 0.48056>	0.61874	0.17247	0.89611	0.4141
A = {1,…,10}	<0.05, 0.525>	0.65536	0.17879	0.905	0.455
A = {1,…,11}	<0.05, 0.56136>	0.6844	0.18331	0.91227	0.4896
A = {1,…,12}	<0.05, 0.59167>	0.70817	0.18665	0.91833	0.5192
A = {1,…,13}	<0.05, 0.61731>	0.72809	0.1892	0.92346	0.545
A = {1,…,14}	<0.05, 0.63929>	0.74513	0.19118	0.92786	0.5677
A = {1,…,15}	<0.05, 0.65833>	0.75993	0.19276	0.93167	0.5877
A = {1,…,16}	<0.05, 0.675>	0.77298	0.19403	0.935	0.6056
A = {1,…,17}	<0.05, 0.68971>	0.78461	0.19508	0.93794	0.6216
A = {1,…,18}	<0.05, 0.70278>	0.79509	0.19595	0.94056	0.6361
A = {1,…,19}	<0.05, 0.71447>	0.80461	0.19668	0.94289	0.6493
A = {1,…,20}	<0.05, 0.725>	0.81333	0.1973	0.945	0.6613

**Table 6.** Description of the datasets [35] used in the experiments.

**Dataset**	**Classes**	**Features**	**Number of Patterns**		

			**For BBAs**	**Training**	**Test**
Yeast	10	8	495	495	494
Glass	6	9	72	71	71
Segment	7	19	770	770	770
Waveform	3	21	1,667	1,667	1,666
Pendigits	10	16	3,664	3,664	3,664

**Table 7.** Description of generating classifiers.

**Dataset**	**Features**	**Feature Distribution**			***k* Value**

		**Classifier 1**	**Classifier 2**	**Classifier 3**	
Yeast	8	1→2	3→5	6→8	15
Glass	9	1→3	4→6	7→9	12
Segment	19	1→10	11→13	14→19	2
Waveform	21	1→8	9→13	14→21	7
Pendigits	16	1→5	6→10	11→16	3

**Table 8.** Correct classification rates of classifiers and two methods.

**Data**	**Yeast**	**Glass**	**Segment**	**Waveform**	**Pendigits**	**Average**
Classifier 1	0.4615	0.6197	0.8558	0.6351	0.7268	**0.6597**
Classifier 2	0.3664	0.6197	0.8636	0.7293	0.8401	**0.6838**
Classifier 3	0.3968	0.6056	0.8896	0.6447	0.8352	**0.6743**
Majority Vote	0.3725	0.7042	0.9104	0.7401	0.8799	**0.7214**
Dempster (No Discounting)	0.4008	0.7183	0.9221	0.7923	0.9427	**0.7552**

**Table 9.** Correct classification rates of five methods using static discounting factor.

**Data**	**Yeast**	**Glass**	**Segment**	**Waveform**	**Pendigits**	**Average**
Elouedi [6]	0.5304	0.7183	0.9351	0.7791	0.9539	**0.7833**
Elouedi(*T_f_*) [9]	0.4615	0.7183	0.9286	0.7809	0.9419	**0.7662**
Yang [8]	0.5385	0.7183	0.9390	0.7815	0.9525	**0.7859**
Guo [2]	0.4595	0.7183	0.9260	0.7809	0.9421	**0.7653**
Our static method	**0.5405**	**0.7324**	**0.9390**	0.7809	0.9531	**0.7892**

**Table 10.** Correct classification rates of three methods using dynamic discounting factor.

**Data**	**Yeast**	**Glass**	**Segment**	**Waveform**	**Pendigits**	**Average**
Guo [2]	0.4352	0.7465	0.9338	0.7665	0.9301	**0.7624**
Xu [12]	0.4352	0.7465	0.9338	0.7725	0.9432	**0.7662**
Our dynamic method	**0.4453**	**0.7465**	0.9312	**0.7773**	**0.9457**	**0.7692**
